# Logarithmic versus Linear Visualizations of COVID-19 Cases Do Not Affect Citizens’ Support for Confinement

**DOI:** 10.1017/S000842392000030X

**Published:** 2020-04-14

**Authors:** Semra Sevi, Marco Mendoza Aviña, Gabrielle Péloquin-Skulski, Emmanuel Heisbourg, Paola Vegas, Maxime Coulombe, Vincent Arel-Bundock, Peter John Loewen, André Blais

**Affiliations:** 1 Department of Political Science, Université de Montréal, Montréal, QC H3T 1N8; 2 Department of Political Science, University of Toronto, Toronto, ON M5S 3K9

## Abstract

The SARS-CoV-2 virus was first identified in Wuhan, China, in late December 2019, and it quickly spread to many countries. By March 2020, the virus had triggered a global pandemic (World Health Organization, 2020). In response to this crisis, governments have implemented unprecedented public health measures. The success of these policies will largely depend on the public's willingness to comply with new rules. A key factor in citizens’ willingness to comply is their understanding of the data that motivate government action. In this study, we examine how different ways of presenting these data visually can affect citizen's perceptions, attitudes and support for public policy.

The SARS-CoV-2 virus was first identified in Wuhan, China, in late December 2019, and it quickly spread to many countries. By March 2020, the virus had triggered a global pandemic (World Health Organization, 2020). In response to this crisis, governments have implemented unprecedented public health measures. The success of these policies will largely depend on the public's willingness to comply with new rules. A key factor in citizens’ willingness to comply is their understanding of the data that motivate government action. In this study, we examine how different ways of presenting these data visually can affect citizen's perceptions, attitudes and support for public policy.

The most common visual strategy to convey information about the spread of COVID-19 is to display a time-series graph of the cumulative number of cases (Figure 1). This type of graph has been used by media and governments all over the world. Indeed, the *New York Times* and the *Financial Times* have entire sections dedicated to updates on COVID-19 cases and deaths in both logarithmic and linear graphs (*Financial Times*, 2020; Katz et al., 2020). The goal of this research note is to understand how these two alternative ways of displaying time-series data affect citizens’ perceptions and beliefs.

**Figure 1.**
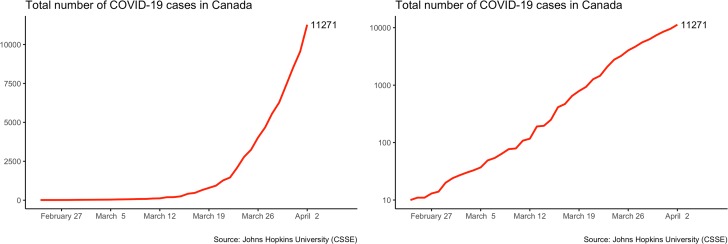
Two Time-Series Plots Showing the Cumulative Number of COVID-19 Cases in Canada Up to April 2, 2020: Left Panel Displays Data on a Linear Scale; Right Panel Displays Data on a Logarithmic Scale

We measure the effect of data visualization on two important outcomes. The first is individuals’ views about the tough measures that have been adopted by authorities. Do Canadians support the confinement policies? The second outcome of interest is pessimism about the prospect of an end to the ongoing crisis. When do Canadians think that they will be allowed to go back to work? These two outcomes are substantively important because they capture the twin goals of public communication: conveying a sense of urgency to people who face a major crisis without making them too pessimistic.

## Time-Series Graphs with Linear or Logarithmic Scales

Many visualization strategies can be used to track the evolution of a public health crisis. During the 2019–2020 pandemic, the most common approach is to plot a simple line to represent the cumulative number of COVID-19 cases over time. Such plots are easy to draw and interpret, but their simplicity can be deceptive. Indeed, data analysts must make several discretionary choices when they draw time-series plots. One important decision concerns the scale on which the variable of interest is displayed.

The default choice for most time-series plots is to use a linear scale on the vertical axis. On that scale, the variable increases *additively* as it rises. In the left panel of Figure 1, we see that the visual distance between 0 and 2500 is exactly the same as the visual distance between 2500 and 5000. These linear graphs are generally considered the simplest and most elementary form of time-series data visualization.

An alternative way to plot time-series data is to use a logarithmic scale on the vertical axis. On that scale, the variable increases *multiplicatively* as it rises. In the right panel of Figure 1, we see that the visual distance between 10 and 100 is the same as the visual distance between 100 and 1000—that is, a 10-fold increase.^1^ The slope of a log-scaled graph measures the relative change in the variable of interest.^2^ This makes it a powerful tool to assess growth rates, which are particularly meaningful in the context of a global health crisis. For instance, the right panel of Figure 1 makes it easy to see that in mid-March 2020, it took just over one week for the number of confirmed COVID-19 cases to increase by a factor of 10. Another reason to use log-scale graphs is that they tend to pull extreme values toward the middle of the distribution. This can be useful when the magnitude of a variable changes dramatically. Finally, log-scale graphs are appropriate when the underlying process that we are modelling is exponential. For example, when the basic reproduction number (R_0_) of an infection is large, the number of cases can increase exponentially. On a log-scale, this exponential increase appears as a straight line, which only bends when the growth rate changes. These properties explain why this visualization strategy is popular in fields like epidemiology and public health economics.

Graphs drawn on a logarithmic scale have many advantages, but it is not clear if ordinary citizens have the numeracy skills to properly interpret them. Most importantly, we do not know what differential effect linear and logarithmic representations of the data have on the perceptions and beliefs of the general public.

As can be seen in Figure 1, the increase in the number of cases with COVID-19 seems much more dramatic when it is displayed on a linear scale because the curve emphasizes the exponential “explosion” of the phenomenon. In contrast, the logarithmic scale shows a straight line with a much flatter slope. Even if the two plots display precisely the same information, citizens may react differently when reading them. Are individuals more supportive of the authorities’ strong public health measures when they see “dramatic” linear plots? Are they more optimistic when they read “tame” logarithmic plots?

## Data and Methods

To answer these questions, we conducted a survey experiment with a sample of 2,500 Canadians interviewed between April 3 and April 5, 2020. This sample was drawn from nationally representative quotas for age, gender, language and province. The sample was provided by Dynata, from their and their partners’ proprietary panels. The survey was hosted on the Qualtrics platform.

Each participant was randomly assigned to one of three groups: a control or one of two treatments. The control group received no information about the outbreak. Each treatment group was shown a time-series plot of the cumulative number of confirmed COVID-19 cases in Canada. The first treatment group saw a linear graph, and the second treatment group saw a logarithmic graph.

Figure 1 shows the two graphs presented to respondents on the first day of the survey.^3^ Those two graphs are quite different. Whereas both display the cumulative number of cases since the beginning of the outbreak, the linear graph (left) conveys a clear a message of crisis: things are worse than before, and the pace is accelerating. In contrast, the smoother trend in the logarithmic graph is much less dramatic. If visual presentation matters, we should observe differences in the experimental groups’ reactions.

Importantly, the two graphs we used are very similar to graphs published in major newspapers, by epidemiologists, and by government agencies. An examination of the *Globe and Mail* (Agius et al., 2020), *Toronto Star* (Tulk et al., 2020) and CBC (CBC, 2020) daily trackers suggests that linear treatments are more common. However, in press conferences held by Ontario and British Columbia on April 3, 2020, and March 27, 2020, respectively, the data were presented on a logarithmic scale (Government of Ontario, 2020; BC Centre for Disease Control, 2020). Accordingly, whereas presenting individuals with a single visual image, embedded in a survey, may seem to be a weak treatment, it closely maps onto how individuals experience and consume similar information when reading a newspaper. The construct validity of our experiment is arguably quite high.

To measure the dependent variables, members of the treatment and control groups were asked two questions. First, we asked them if they support or oppose the governments’ instruction to remain at home (scale of 0 to 10). Second, we asked respondents when they expect the government to allow nearly everyone back to work.^4^ Complete question wordings are reported in the online appendix.

The main question that we address is whether it makes a difference if individuals see graphs drawn on a linear or a logarithmic scale. Those who are shown the logarithmic scale see a slope that is less steep than those who are shown the linear scale. As a result, the former group may perceive the risk from contagion to be less serious.

## Results

**Figure 2.**
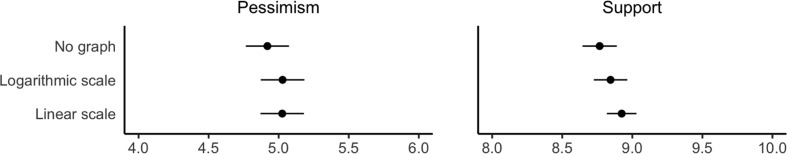
Mean Pessimism and Support in the Control and Treatment Groups

Our findings are easy to summarize. For both dependent variables—pessimism and support— there is essentially no difference between the two treatment groups and also essentially no difference between the treatment groups and the control group. Figure 2 shows that presenting a graph or not does not make a difference and also that people react the same way whether they are shown a graph with a logarithmic or a linear scale. We discuss the implications of these results below.

Before doing so, it is important to note the extraordinary support for confinement. Fifty-four per cent of respondents say they fully support the stringent measures that force most people to stay home (score of 10, on a 0 to 10 scale). Furthermore, a strong majority of Canadians believe that confinement will have to last at least two more months; only 10 per cent of respondents expect most people to come back to work in April or May.^5^

Do citizens’ reactions vary across socio-demographic groups? The mortality rate is much higher among the eldest, so they may be particularly supportive of confinement measures, especially given those who are retired and therefore are not prevented from working. Likewise, women are generally more supportive of social protection measures (Gidengil, 1995), and they may be more willing to accept the necessity of confinement. Similarly, the more educated may be more strongly exposed to the unanimous elite message that radical measures are needed (Zaller, 1992). Finally, the severity of the contagion, as well as the way the authorities communicate with the public, varies substantially across provinces. So there may be variation across regions.

**Figure 3.**
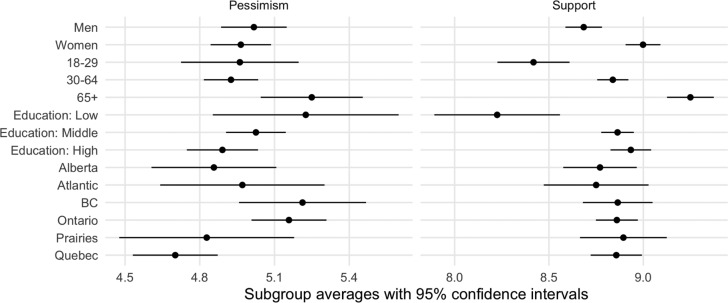
Mean Pessimism and Support by Region, Gender, Education and Age

Again, our findings are basically null, as can be seen in Figure 3 (see online appendix for regression results). Canadians’ reactions vary little by age, gender, education or region. Most of the time, the differences between the groups are not statistically significant (despite the fact that we have a big sample), and where they are, the magnitude of the effect is quite small. The eldest are more supportive of confinement, but the youngest are also strongly in favour. Quebeckers are slightly more optimistic about when they will be able to come back to work, but the absence of regional difference in support for confinement is even more striking.

We interpret these findings as indicators of the stunning success of the public health experts and governments in convincing citizens that the measures taken are necessary and may have to last for at least some months. These are neither “nice” nor “easy” messages. People are inclined to resist such unpleasant messages. But Canadians are accepting elite messages and the bad news.

There are two possible reasons why our treatments did not have any effect. The first is that Canadians have already formed strong and firm opinions on the issue; they have come to believe that this is a serious crisis and that the authorities are doing what is necessary. Providing them with differing visual displays of information has no effect on these views. The second is that they have already been widely exposed to both linear and logarithmic graphs before our experiment, and as a consequence, our treatment did not convey any new information; people have been conditioned by “pretreatment” from events outside the experiment (Druckman and Leeper, 2012). We are unable to tell which of these two reasons applies in the present case. This is a reminder, however, that the null finding observed in this study cannot necessarily be generalized to all contexts.

## Notes

1 Analysts can use logarithms with a different base. For instance, values on the *y*-axis could increase by multiples of 2 instead of 10.

2 A mathematical identity ensures that the difference between the log number of cases at time *t* and the log number of cases at time *t-1* is equal to the log of the ratio between those two quantities: .

3 Graphs were updated daily using data from Dong et al. (2020).

4 This outcome variable is coded as: 1-April, 2-May, 3-June, 4-July, 5-August, 6-September, 7-October, 8-November, 9-December, 10-Sometime in 2021, 11-Never.

5 We must acknowledge the possibility that some respondents without clear opinion on the issue may have been inclined to choose the middle options. See the online appendix for the distribution of the variables by treatment.

## Supplementary material

For supplementary material accompanying this paper visit http://dx.doi.org/10.1017/S000842392000030X. click here to view supplementary material
