# Supplementary material for: Logarithmic versus Linear Visualizations of COVID-19 Cases Do Not Affect Citizens’ Support for Confinement
Source: Can J Polit Sci. 2020 Apr 14:1–6. doi: 10.1017/S000842392000030X (PMC7200843; doi:10.1017/S000842392000030X)
Supplement: Supplementary file 1 [file S000842392000030Xsup001.docx]

**Appendices**

**Exact wording of the questions:**

Because of the COVID-19 epidemic, governments are asking everyone to stay home, except for strictly essential work and basic necessities like grocery shopping. How strongly do you support or oppose that decision? (0 to 10 scale; I fully oppose to I fully support)

When do you think that governments will allow nearly everyone to go back to work?

April 2020

May 2020

June 2020

July 2020

August 2020

September 2020

October 2020

November 2020

December 2020

Sometime in 2021

Never

**Example of question wording for the two treatments**

Here is a graph showing the evolution of COVID-19 cases in Canada:


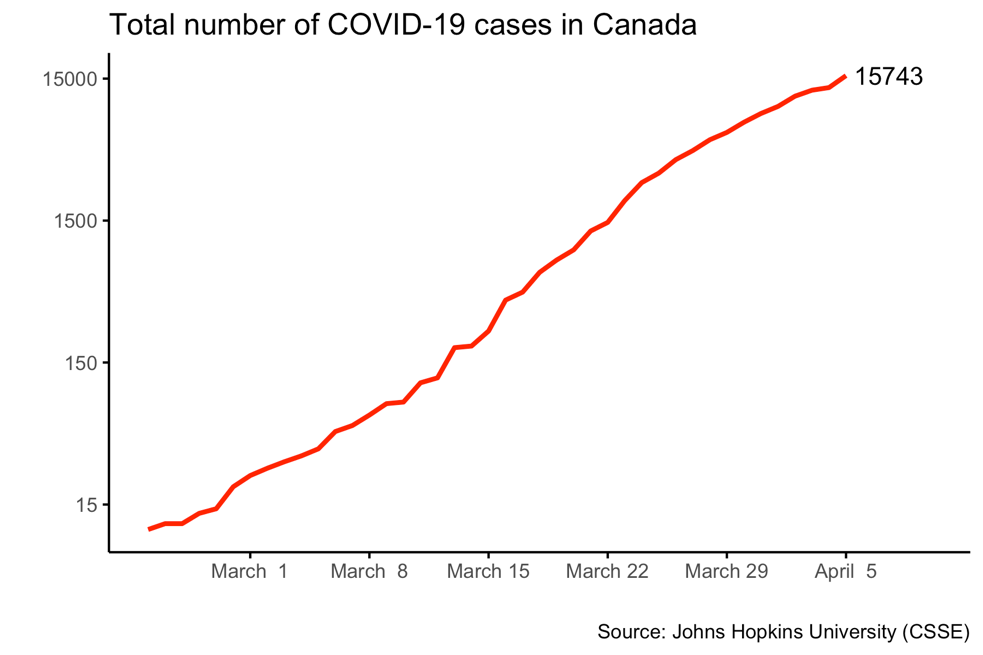


Here is a graph showing the evolution of COVID-19 cases in Canada:


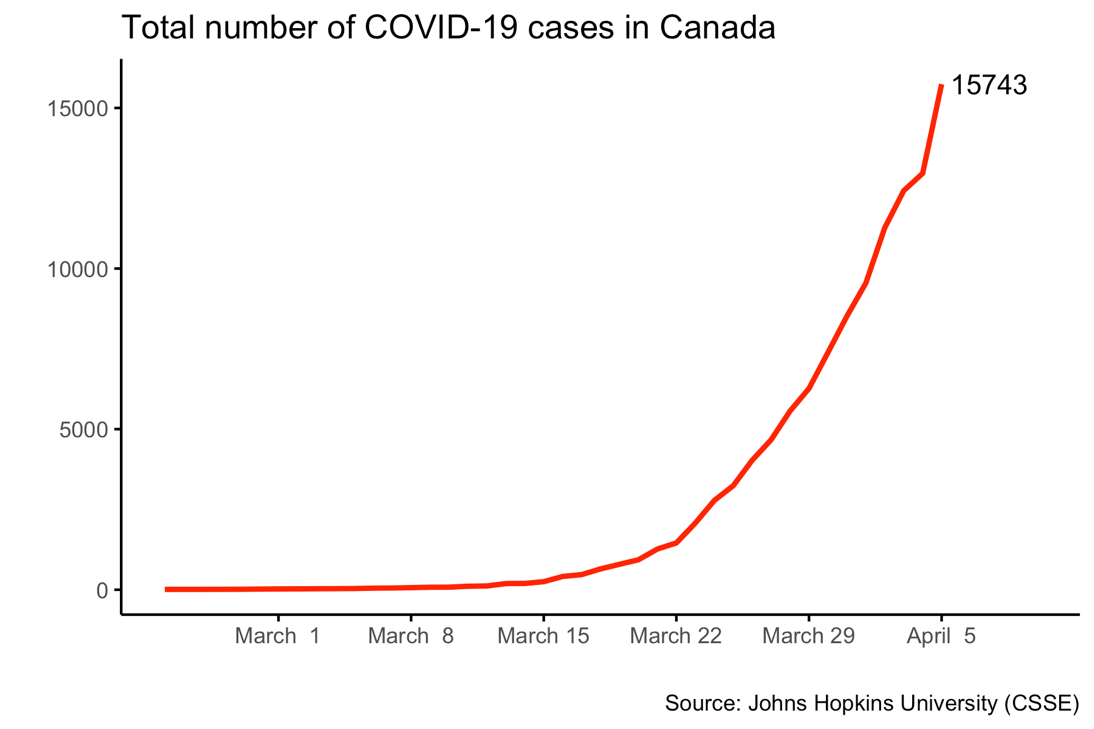


**Distribution of the dependent variables**

Figure 1: Distribution of responses for the pessimism and support variables


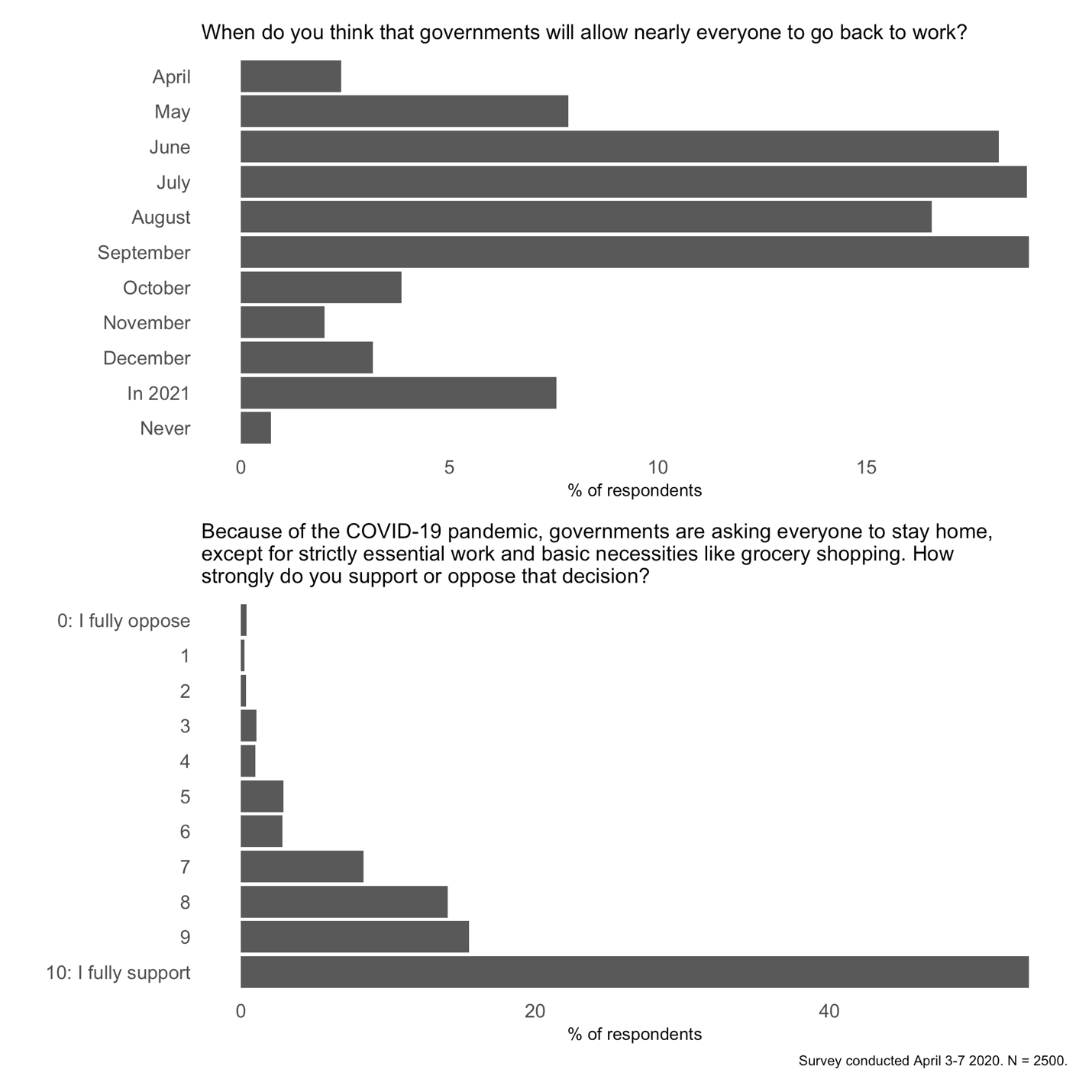


Figure 2: Distribution of responses for the pessimism and support variables by treatment


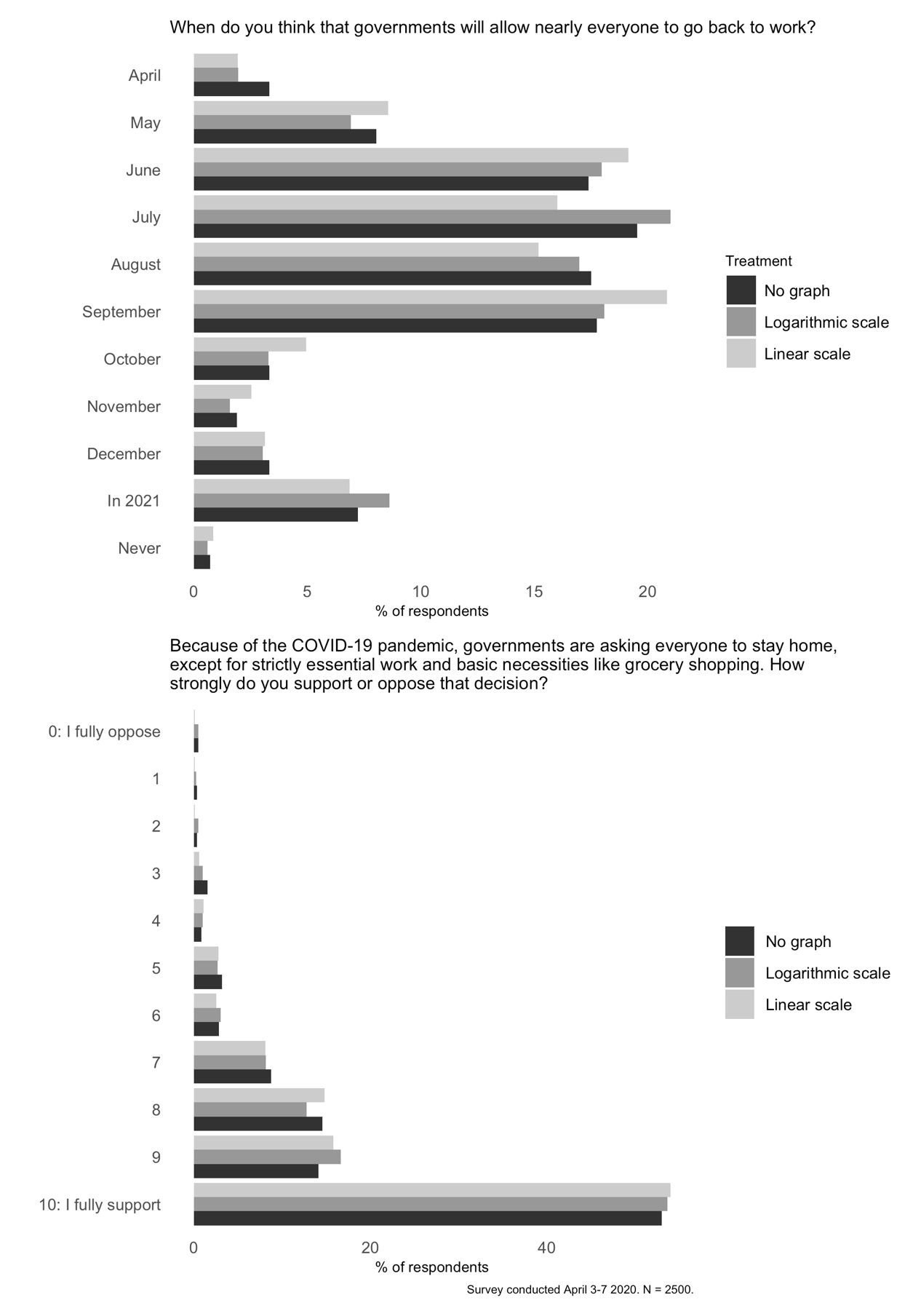


**Full regression results**

Table 1. Full results (pessimism) by experimental condition, age group, region, and gender (OLS regression)

|  | Lin vs. Log | Lin or Log | Gender | Age | Education | Region |
| --- | --- | --- | --- | --- | --- | --- |
| Linear scale | 0.106 |  |  |  |  |  |
|  | (0.111) |  |  |  |  |  |
| Logarithmic scale | 0.108 |  |  |  |  |  |
|  | (0.112) |  |  |  |  |  |
| Either Graph |  | 0.107 |  |  |  |  |
|  |  | (0.096) |  |  |  |  |
| Women |  |  | -0.052 |  |  |  |
|  |  |  | (0.091) |  |  |  |
| 30-64 |  |  |  | -0.036 |  |  |
|  |  |  |  | (0.126) |  |  |
| 65+ |  |  |  | 0.289 |  |  |
|  |  |  |  | (0.155) |  |  |
| Education: Low |  |  |  |  | 0.335 |  |
|  |  |  |  |  | (0.189) |  |
| Education: Middle |  |  |  |  | 0.135 |  |
|  |  |  |  |  | (0.097) |  |
| Quebec |  |  |  |  |  | -0.155 |
|  |  |  |  |  |  | (0.166) |
| Atlantic |  |  |  |  |  | 0.114 |
|  |  |  |  |  |  | (0.220) |
| Ontario |  |  |  |  |  | 0.301 |
|  |  |  |  |  |  | (0.155) |
| Prairies |  |  |  |  |  | -0.029 |
|  |  |  |  |  |  | (0.217) |
| BC |  |  |  |  |  | 0.356 |
|  |  |  |  |  |  | (0.184) |
| Intercept | 4.920 | 4.920 | 5.017 | 4.961 | 4.891 | 4.857 |
|  | (0.078) | (0.078) | (0.065) | (0.112) | (0.076) | (0.136) |
| Num.Obs. | 2499 | 2499 | 2499 | 2499 | 2499 | 2499 |
| R2 | 0.000 | 0.000 | 0.000 | 0.003 | 0.002 | 0.008 |
| Adj.R2 | -0.000 | 0.000 | -0.000 | 0.002 | 0.001 | 0.006 |
| AIC | 11210.7 | 11208.7 | 11209.6 | 11204.6 | 11208.0 | 11198.4 |
| BIC | 11234.0 | 11226.2 | 11227.1 | 11227.9 | 11231.3 | 11239.1 |
| Log.Lik. | -5601.356 | -5601.356 | -5601.809 | -5598.309 | -5600.017 | -5592.179 |

Table 2: Full results (support) by experimental condition, age group, region, and gender (OLS regression)

|  | Lin vs. Log | Lin or Log | Gender | Age | Education | Region |
| --- | --- | --- | --- | --- | --- | --- |
| Linear scale | 0.157 |  |  |  |  |  |
|  | (0.083) |  |  |  |  |  |
| Logarithmic scale | 0.078 |  |  |  |  |  |
|  | (0.084) |  |  |  |  |  |
| Either Graph |  | 0.118 |  |  |  |  |
|  |  | (0.072) |  |  |  |  |
| Women |  |  | 0.314 |  |  |  |
|  |  |  | (0.068) |  |  |  |
| 30-64 |  |  |  | 0.420 |  |  |
|  |  |  |  | (0.093) |  |  |
| 65+ |  |  |  | 0.831 |  |  |
|  |  |  |  | (0.115) |  |  |
| Education: Low |  |  |  |  | -0.708 |  |
|  |  |  |  |  | (0.141) |  |
| Education: Middle |  |  |  |  | -0.071 |  |
|  |  |  |  |  | (0.072) |  |
| Quebec |  |  |  |  |  | 0.086 |
|  |  |  |  |  |  | (0.125) |
| Atlantic |  |  |  |  |  | -0.021 |
|  |  |  |  |  |  | (0.166) |
| Ontario |  |  |  |  |  | 0.089 |
|  |  |  |  |  |  | (0.116) |
| Prairies |  |  |  |  |  | 0.124 |
|  |  |  |  |  |  | (0.163) |
| BC |  |  |  |  |  | 0.094 |
|  |  |  |  |  |  | (0.138) |
| Intercept | 8.767 | 8.767 | 8.684 | 8.418 | 8.934 | 8.771 |
|  | (0.059) | (0.059) | (0.049) | (0.084) | (0.056) | (0.102) |
| Num.Obs. | 2499 | 2499 | 2499 | 2499 | 2499 | 2499 |
| R2 | 0.001 | 0.001 | 0.008 | 0.021 | 0.010 | 0.001 |
| Adj.R2 | 0.001 | 0.001 | 0.008 | 0.020 | 0.009 | -0.001 |
| AIC | 9769.6 | 9768.5 | 9749.9 | 9721.4 | 9747.6 | 9777.8 |
| BIC | 9792.9 | 9786.0 | 9767.4 | 9744.7 | 9770.9 | 9818.6 |
| Log.Lik. | -4880.811 | -4881.259 | -4871.960 | -4856.698 | -4869.821 | -4881.909 |
